# 免疫介导的再生障碍性贫血模型小鼠巨噬细胞归巢及特征分析

**DOI:** 10.3760/cma.j.cn121090-20230927-00142

**Published:** 2024-06

**Authors:** 玮 孙, 赠华 林, 晗 王, 惠 贾, 来根 童, 志鹏 张, 玟 李, 诚程 周, 红 刘

**Affiliations:** 1 南通大学医学院，南通 226001 Medical School, Nantong University, Nantong 226001, China; 2 南通大学附属医院，南通 226001 Affiliated Hospital of Nantong University, Nantong 226001, China; 3 宜兴市人民医院，宜兴 214200 Yixing People's Hospital, Yixing 214200, China

## Abstract

研究免疫介导的再生障碍性贫血（AA）模型小鼠体内巨噬细胞在不同器官的动态归巢过程、特征。通过磁珠分选出供鼠淋巴结中巨噬细胞并用PKH67荧光标记，参照AA模型制备方法造模后，分析小鼠血常规、骨髓活检及HE染色结果，验证造模效果。在造模的第4、8、12天，收集骨髓、脾脏和淋巴结单个核细胞，通过流式细胞术分析PKH67荧光标志供鼠巨噬细胞的动态变化。探究PKH67荧光标志的巨噬细胞，在AA模型小鼠发病过程中的动态变化，观测到供鼠巨噬细胞归巢到淋巴结并扩增分化，最终转运至骨髓和脾脏。通过蛋白组学质谱分析，初步揭示了巨噬细胞参与AA骨髓微环境激活后的相关免疫炎症反应通路，为病理性巨噬细胞参与AA模型小鼠的发病提供了依据。

## Abstract (en)

To investigate the dynamic homing process and characteristics of macrophages in different organs of immune-mediated aplastic anemia（AA）model mice. Macrophages in donor lymph nodes were sorted by magnetic beads and labeled with PKH67. After modeling according to the preparation method of the AA model, peripheral blood rountine analysis, bone marrow biopsy and HE staining results were analyzed to verify the modeling effect. On days 4, 8, and 12 of modeling, the bone marrow, spleen, and lymph node mononuclear cells were collected, and dynamic changes of PKH67-labeled macrophages in donor mice were analyzed by flow cytometry. In this study, dynamic changes in PKH67-labeled macrophages in the pathogenesis of AA model mice were explored. Macrophages in donor mice homed to the lymph nodes, expanding and differentiating in the lymph nodes, and finally transported to the bone marrow and spleen. Through proteomics mass spectrometry analysis, the related immune inflammatory response pathway of macrophages involved in the activation of the AA bone marrow microenvironment was preliminarily revealed, which provides a basis for the pathological macrophages involved in the pathogenesis of AA model mice.

再生障碍性贫血（AA）是一种罕见但严重的造血系统疾病，其特点是骨髓造血功能减退，导致三系细胞减少，进而引起贫血、感染和出血等症状[1]。细胞毒性T细胞介导的骨髓造血功能衰竭和自身免疫导致的造血功能受损，在获得性AA发病过程中占主导地位，同时调节性T（Treg）细胞功能缺陷也参与了AA的发病[2]。标准的免疫抑制治疗有效率为60％～65％，提示细胞毒性T细胞以外的因素也参与了AA的发病[3]。

Sun等[4]通过敲除AA模型小鼠TNF-α发现，巨噬细胞通过TNF-α刺激细胞毒性T细胞分泌大量IFN-γ，在AA中加速骨髓衰竭。我们先前的实验证实，在AA模型小鼠疾病的终末期归巢到骨髓的淋巴细胞具有很强的免疫攻击活性，而其免疫抑制能力则大幅下降[5]。本研究运用自行改良的适合本实验条件的B6D2F1（以下简称F1）模型小鼠[6]，选用预染供鼠巨噬细胞混合供鼠淋巴细胞悬液和全身照射（total body irradiation，TBI）联合的方法，建立可以在骨髓、脾脏、淋巴结内追踪供鼠巨噬细胞归巢过程的AA模型小鼠，探讨供鼠巨噬细胞在AA发病过程中的动态变化及其对骨髓微环境的影响，以进一步阐明AA的发病机制。

## 材料与方法

1. 实验研究动物：父代雄性DBA/2（小鼠编号：214）和母代雌性C57BL/6N（B6，小鼠编号：213）均购自北京维通利华实验动物技术有限公司，实验用雌性F1小鼠（小鼠编号：302，8周龄，体重18～22 g），为DBA/2与B6小鼠配种后的子一代雌性小鼠。采用随机数字表法将雌性F1小鼠随机分为对照组（4只）、AA组（16只）。所有实验小鼠代养于南通大学医学院实验动物中心。本研究所用实验动物均经南通大学医学院动物实验管理委员会批准。

2. 实验试剂及仪器：PKH67 Green Fluorescent Cell Linker Kit（西格玛奥德里奇上海有限公司），用于常规细胞膜标记；IMDM培养基、RPMI1640培养基、PBS缓冲液（美国Gibco公司）；无菌离心管、流式管、EP管、细胞培养皿等耗材（无锡耐思生命科技有限公司）；抗小鼠CD3-FITC、CD4-PerCP-Cy5.5、CD8-APC、F4/80-BV421、CD11b-BV510、CD86-PE-cy7、CD206-Alexa647、CD25-APC、FOXP3-PE抗体、Pharmingen固定/破膜试剂盒、流式细胞仪（美国BD公司），Anti-F4/80 MicroBeads UltraPure（德国美天旎生物科技公司），全自动血细胞分析仪（日本希森美康公司），CLINAC-TRILOGY直线加速器（美国VARIAN公司）。5D非标记定量蛋白质组学分析技术及相关试剂、耗材、设备由上海吉凯基因生物科技有限公司提供。

3. 磁珠分选后预染巨噬细胞及AA模型小鼠的建立：取B6小鼠（与F1小鼠同周龄同性别），麻醉后无痛处死，在超净台下取B6小鼠全身浅表淋巴结，置于RPMI 1640培养基中，经研磨器研磨过滤，制成单个核细胞悬液。调整细胞混悬液浓度为（1.9～2.1）×10^7^/ml冰上备用。分别取上述细胞悬液，按照说明进行磁珠分选，收集F4/80^+^细胞，并用PKH67细胞膜染色剂进行染色；染色后的巨噬细胞与原F4/80^+^磁珠分选后的细胞悬液混合，标记为AA组。参照AA模型制备方法[6]，模型小鼠制作完成后放回原处饲养，造模当天为第0天，第12天采集AA模型小鼠（4只）眼眶静脉血、股骨骨髓单个核细胞（BMMNC）以及胸骨、股骨、脾脏组织HE染色，分析组间小鼠的外周血常规、BMMNC计数以及HE染色等病理结果，验证造模效果。取剩余小鼠骨髓、脾脏、淋巴结，制备成单细胞悬液备用。

4. 供鼠来源巨噬细胞动态变化监测：在输注PKH67荧光标志的巨噬细胞与淋巴结单个细胞的混悬液的第4、8、12天，分3次每次处死4只AA模型小鼠，收集淋巴结、脾脏以及骨髓样本制备成单个核细胞悬液，通过流式细胞术分析标本中供鼠来源的PKH67巨噬细胞的动态变化。

5. 骨髓和脾脏CD4^+^/CD8^+^ T淋巴细胞、Treg、巨噬细胞的流式细胞术检测：取100 µl BMMNC悬液，加入CD3（FITC）、CD4（PerCP-Cy5.5）和CD8（APC）抗体，孵育35 min。加入流式染色缓冲液，离心，弃上清，用Pharmingen固定/破核试剂盒方法破核。加入FOXP3（PE），孵育50 min，清洗2次，再加入流式染色缓冲液。进行流式细胞仪检测。巨噬细胞及其亚型的检测：取100 µl脾脏或BMMNC悬液，加入CD16/32抗体进行阻断。进行细胞表面染色，加入F4/80（Percp-Cy5.5）、CD11b（FITC）、CD45（APC）和CD86（PE-cy7）抗体，孵育30 min。清洗后，用Pharmingen固定/破膜试剂盒方法破膜。加入CD206（Alexa647）进行胞内染色，孵育45 min，洗涤后上流式细胞仪检测。

6. 非标记定量蛋白质组学分析：在输注淋巴细胞联合TBI照光后的第12天，收集AA组和对照组小鼠的骨髓单个核细胞悬液，通过对样本蛋白质抽提，定量与质检，FASP酶解，质谱检测通过后，上机检测，分析数据。

7. 统计学处理：采用GraphPad Prism 8.0.2、FlowJo_v10.8.1、Perseus 1.3（Max Planck Institute of Biochemistry in Martinsried，Germany）、PaSER2023、Rversion 3.3.1、Adobe Illustrator 2023软件进行绘图、蛋白组学质谱分析以及描述性统计学分析，每组数据均来自3次及以上独立实验，两组间比较采用双尾非配对*t*检验，多组间比较采用单因素方差分析。结果以*x*±*s*表示，*P*<0.05为差异具有统计学意义。

## 结果

一、B6小鼠来源的淋巴细胞、巨噬细胞诱导B6D2F1模型小鼠的建立和骨髓流式细胞术及组织病理学鉴定

1. 造模后第12天，AA组与对照组相比外周血WBC、HGB、PLT、ANC、BMMNC计数均明显低于对照组（图1）。骨髓HE染色示，AA组小鼠髓腔内造血组织减少，呈脂质空泡状、骨髓空虚。脾脏HE染色示，AA组脾脏红白髓分界不清，白髓中淋巴小结生发中心消失，脾窦腔内空虚、血细胞减少（图2）。

**图1:**
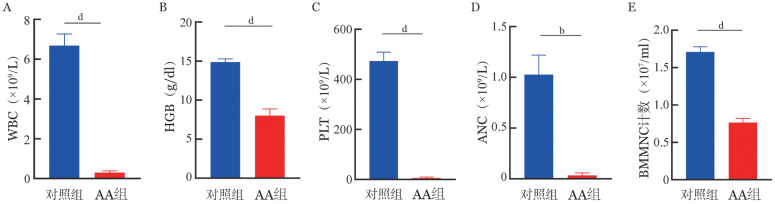
B6小鼠来源的淋巴细胞、巨噬细胞诱导F1模型小鼠外周血WBC（A）、HGB（B）、PLT（C）、ANC（D）、BMMNC计数（E）结果 **注** 对照组：正常F1小鼠；AA组：再生障碍性贫血模型小鼠；BMMNC：股骨骨髓单个核细胞；*P*值：^b^*P*<0.01、^d^*P*<0.0001

**图2:**
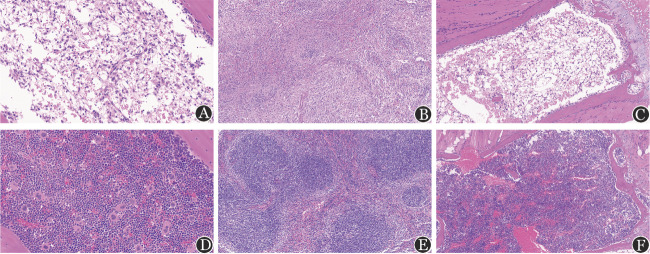
再生障碍性贫血模型小鼠与正常F1小鼠的组织病理结果（×40，HE染色） **A～C** 再生障碍性贫血模型小鼠的股骨、胸骨、脾脏；**D～F** 正常F1小鼠的股骨、胸骨、脾脏

2. F1AA小鼠在第12天骨髓CD4^+^ T细胞、CD8^+^T细胞、Treg细胞、M_1_型巨噬细胞、M_2_型巨噬细胞以及总巨噬细胞对照组与AA组比较：见表1，与对照组相比AA模型小鼠骨髓中CD8^+^/CD4^+^T细胞比例增高，Treg细胞比例下降；骨髓中M_1_、M_2_型以及总巨噬细胞占比，M_1_型、M_2_型巨噬细胞比例在AA组中明显上升；总巨噬细胞占比与对照组相比上升且组内与组间比较差异有统计学意义（*P*值均<0.05）。

**表1:** 对照组与AA组小鼠骨髓淋巴细胞亚群、Treg细胞、巨噬细胞亚群及总巨噬细胞比较（*x*±*s*，％）

细胞比例	对照组（3只）	AA组（3只）	*P*值
CD4^+^细胞	0.98±0.27	37.8±0.46	<0.0001
CD8^+^细胞	1.42±0.61	58.83±1.58	<0.0001
Treg细胞	3.87±1.64	0.22±0.11	<0.05
M_1_巨噬细胞	16.90±1.55	76.40±3.12	<0.0001
M_2_巨噬细胞	7.10±1.17	16.90±1.55	<0.001
总巨噬细胞	9.31±1.57	24.3±6.95	<0.05

**注** 对照组：正常F1小鼠；AA组：再生障碍性贫血模型小鼠

二、PKH67巨噬细胞在不同器官的归巢过程分析

供鼠来源巨噬细胞在不同器官的分布：如图3所示，AA模型小鼠体内，骨髓和脾脏中PKH67荧光标志的巨噬细胞在巨噬细胞群中比例随时间逐渐增加，淋巴结内PKH67荧光标志的巨噬细胞在巨噬细胞群中比例随时间逐渐减少；在第4天，AA模型小鼠骨髓、脾脏和淋巴结中PKH67荧光标志的巨噬细胞比例分别为（0.79±0.1）％、（1.49±0.44）％、（54.57±7.2）％；在第8天比例分别为（2.59±0.87）％、（5.61±0.47）％、（24.9±4.35）％；而在第12天时，PKH67荧光标志的巨噬细胞在骨髓和脾脏中的比例高于淋巴结中的比例，分别为（6.97±1.37）％、（21.23±1.79）％、（5.82±1.37）％，组内及组间两两比较差异均有统计学意义（*P*均<0.05）（图3）。

**图3:**
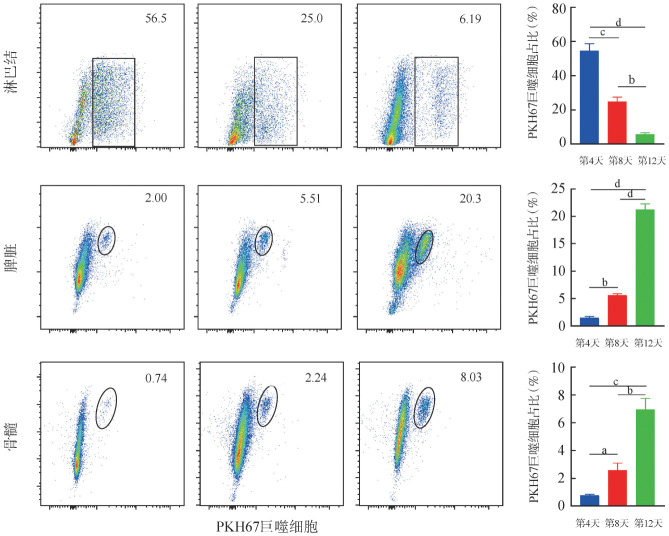
流式细胞术检测不同时间F1再生障碍性贫血模型小鼠体内PKH67荧光标志的巨噬细胞在不同器官内的分布 **注** ^a^*P*<0.05、^b^*P*<0.01、^c^*P*<0.001、^d^*P*<0.0001

三、5D Labelfree蛋白质组学质谱分析

组学生物进程的GO富集分析显示，与巨噬细胞活化通路相关蛋白NR1D2、SLC11A1、NFKBIA、ADIPOQ、C1QC表达明显上调；与巨噬细胞活化和分化通路相关蛋白GATA1、GATA2、ABCG4、PTPN2、THOC5、SLC11A2、FOXP1、FOXP4、ITGB3、ITGB5、ZBTB44表达下调（图4A）；生物进程途径分析显示，T细胞分化、巨噬细胞活化及造血细胞谱系相关的蛋白上调（图4B）；在通路富集分析中观察到移植物抗宿主病、细胞周期、Th1、Th2及Th17细胞分化等通路明显上调（图4C）。

**图4:**
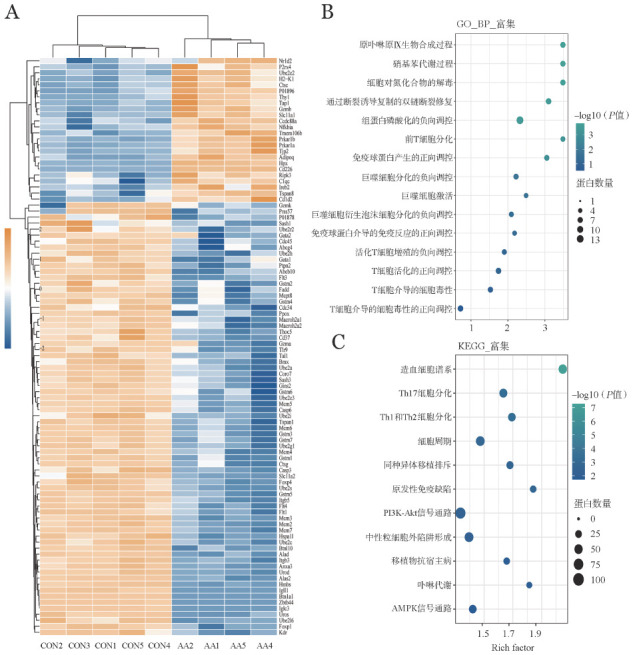
免疫介导的再生障碍性贫血模型小鼠与对照组小鼠骨髓来源的单个核细胞非标记定量蛋白质组学分析聚类图（A）、生物进程（B）及KEGG通路富集分析（C） **注** CON：正常F1小鼠；AA：再生障碍性贫血模型小鼠

## 讨论

越来越多的研究发现骨髓造血微环境对于造血干细胞的影响也很重要，巨噬细胞是骨髓微环境的重要组成部分[7]。在复杂的微环境中表现出多样化的功能和行为，可分为M_1_型和M_2_型巨噬细胞，M_1_型巨噬细胞分泌促炎因子和趋化因子，可将抗原提呈，参与免疫应答；M_2_型巨噬细胞主要参与抗炎和免疫调节作用[8]–[9]。有学者在动物模型中验证了巨噬细胞及其亚型与AA的关系，指出M_1_型巨噬细胞参与了AA的发病[10]，且临床上接受造血干细胞移植的患者骨髓中巨噬细胞、细胞毒性T细胞增加，常预示异基因骨髓移植预后较差，证实了巨噬细胞抑制造血的作用[11]。

“细胞归巢（cell homing）”通常是指一种生物学过程，其中特定类型的细胞离开其当前位置，移动到特定的目标组织或器官，并在那里发挥其功能，此过程在发育、免疫响应和组织修复中都非常重要[12]。在某些情况下，巨噬细胞可以在外周血或其他组织中增多，并通过归巢的过程，回到骨髓。巨噬细胞的归巢机制通常涉及细胞表面受体、趋化因子和细胞黏附分子的相互作用，以及细胞对化学信号和梯度的感应等一系列的复杂过程，这些机制确保细胞能够准确地移动到它们需要的位置，并在那里执行其特定的功能[13]。本研究用PKH67荧光标志B6巨噬细胞，注射到F1小鼠体内，通过流式细胞术对巨噬细胞进行示踪，探究了AA小鼠发病过程中PKH67荧光标志的巨噬细胞在不同器官的归巢特征。实验结果显示该模型的发病时间和既往正常的B6小鼠淋巴结单个细胞诱导的F1模型的发病时间一致，在第12天时外周血象及股骨有核细胞计数均显著下降，骨髓和脾脏的病理学结果符合AA小鼠骨髓衰竭的病理表现。在模型中我们观察了不同时间点由PKH67荧光标志的供鼠巨噬细胞在模型小鼠体内的分布，结果显示在骨髓衰竭的早期阶段，F1小鼠骨髓和脾脏中的PKH67荧光标志的巨噬细胞比例非常低，而在淋巴结中的比例相对较高。在骨髓衰竭后期骨髓中的PKH67荧光标志的巨噬细胞比例显著升高，由此我们推测来源于淋巴组织的供鼠巨噬细胞在进入宿主小鼠体内后，首先归巢到外周淋巴器官，在其中扩增并活化，然后迁移至骨髓和脾脏，参与AA的发病。因而，我们推测免疫介导的AA模型小鼠巨噬细胞在骨髓衰竭过程中具有活化和归巢的能力，这与过去一项在免疫型AA模型小鼠体内示踪DsRed小鼠病理性T细胞的研究有相似之处[5]，而巨噬细胞参与AA的机制及与T细胞之间的交互作用尚待进一步研究。

在骨髓衰竭晚期阶段，我们提取AA组小鼠与对照组小鼠的骨髓蛋白进行蛋白质谱分析。在通路富集分析中观察到移植物抗宿主病、细胞周期变化、Th1、Th2及Th17细胞分化等通路明显上调，细胞分化过程的失调，可导致免疫系统的异常激活，影响造血功能。通过组学生物进程的GO富集分析，与巨噬细胞活化通路相关蛋白NR1D2、SLC11A1、NFKBIA、ADIPOQ、C1QC表达升高，其主要与适应性免疫、巨噬细胞活化、CD8^+^T细胞免疫活性及免疫复合物清除、调节巨噬细胞对骨髓微环境的免疫应答等有关[14]–[16]。与此相反，与巨噬细胞活化和分化通路相关蛋白GATA1、GATA2、ABCG4、PTPN2、THOC5、SLC11A2、FOXP1、FOXP4、ITGB3、ITGB5、ZBTB44表达减低，主要与细胞免疫抑制能力下降、造血干细胞分化增殖受限、粒细胞-巨噬细胞功能受限、巨噬细胞过度激活等有关[17]–[21]。生物进程途径分析表明，T细胞分化、巨噬细胞活化及造血细胞谱系相关蛋白上调，异常活化的巨噬细胞可分泌促炎症因子，促进骨髓内细胞毒性T细胞分泌大量干扰素-γ从而导致造血干细胞凋亡，诱发骨髓衰竭[4]。随着骨髓中巨噬细胞的增多，免疫诱导的AA小鼠中供鼠巨噬细胞可引起骨髓衰竭相应的微环境变化，但同时也不能排除早期归巢到骨髓少量的PKH67荧光标志的M_1_和M_2_型巨噬细胞的作用，自身在骨髓内由于异体抗原刺激下不断扩增引起细胞比例增加。本研究仅观测到模型小鼠骨髓微环境内自身巨噬细胞亚型的分化，尚未观察到PKH67荧光标志的巨噬细胞在AA模型小鼠发病过程中的亚型变化。由于该实验基于AA模型小鼠的研究，观测到宿主淋巴结中的PKH67荧光标志的巨噬细胞大部分转移至骨髓和脾脏组织，其机制是否与AA患者发病过程相同，尚待进一步证实。

总之，本研究建立了可以在不同器官中追踪PKH67荧光标志的巨噬细胞归巢过程的免疫介导的AA模型小鼠，并对PKH67荧光标志的巨噬细胞归巢特征进行分析，证实了异体巨噬细胞在AA发病过程中的动态变化，并探讨了巨噬细胞与AA发病的关系。同时我们通过提取AA小鼠骨髓单个核细胞蛋白进行蛋白质谱分析，初步揭示了巨噬细胞参与AA骨髓微环境激活后的相关免疫炎症反应通路，为病理性巨噬细胞参与AA模型小鼠的发病提供了依据。
